# Enhancing Phenolic Content of Medicinal Aromatic Plants Extracts-Biofunctional Foods Preparation

**DOI:** 10.3390/plants11010076

**Published:** 2021-12-27

**Authors:** Maria G. Ziagova, Charoula Mavromatidou, Georgios Samiotis, Elisavet Amanatidou

**Affiliations:** Laboratory of Environmental Chemistry & Water and Wastewater Treatment, Department of Chemical Engineering, University of Western Macedonia, Koila, 50100 Kozani, Greece; waterlab1@uowm.gr (M.G.Z.); waterlab3@uowm.gr (C.M.); waterlab2@uowm.gr (G.S.)

**Keywords:** medicinal and aromatic plants, chocolate biofunctional foods, polyphenol encapsulation, *Melissa officinalis* antioxidants, gum arabic encapsulating material, inulin encapsulating material, natural sweetener erythritol, natural sweetener honey

## Abstract

In this study, the assessment of TPC and antioxidant activity enhancement of medicinal and aromatic plant (MAP) aqueous extracts using natural sweeteners or encapsulation materials was carried out. MAP extracts fortified with polyphenols were used to produce biofunctional chocolate bites. Honey or erythritol added to *Melissa officinalis* concentrated aqueous extracts exhibited TPC at 19.53 mg GAE/mL and 18.24 mg GAE/mL, respectively, and DPPH radical scavenging activity greater than 82%, comparing to its non-concentrated aqueous extract (3.74 mg GAE/mL and 72.9%, respectively). Honey added to MAP concentrated aqueous extract mixtures presented up to twofold higher TPC compared to *M. officinalis* concentrated aqueous extracts with honey. Chocolate bites with MAP concentrated aqueous extract mixtures and honey exhibited TPC and DPPH radical scavenging activity at 29.48 mg GAE/g chocolate and 93.7%, respectively. The addition of gum arabic or inulin in MAP concentrated aqueous extract mixtures increased the TPC up to 12-fold (40.37 mg GAE/mL and 34.14 mg GAE/mL, respectively) compared to its non-concentrated aqueous extracts (3.38 mg GAE/mL), whereas DPPH radical scavenging activity approached 99.5%. Honey incorporation as a sweetener and polyphenolic compound encapsulation in gum arabic can lead to the production of biofunctional foods with elevated cytoprotective action without compromising their organoleptic attributes.

## 1. Introduction

The increasing demand for natural compounds, following the concerns about the side effects of synthetic compounds, has greatly promoted the use of plant extracts as functional ingredients in the food and pharmaceutical industries. Among plant-derived molecules, polyphenols represent an attractive class of bioactive molecules because of their multiple actions and benefits to human health, ascribed essentially to their high antioxidant activity [1]. Medicinal aromatic plants (MAPs) such as lemon balm (*Melissa officinalis* L.) are widely used in traditional medicine and cuisine due to the high amounts of polyphenols they contain, possessing also several other beneficial properties, such as antioxidant, antimicrobial, and antitumor effects [2]. Therefore, MAP extracts are considered safe to be used as food supplements in healthy individuals, also providing relief for heart benign palpitation in humans, confirming the high interest in these plants [3]. The phenolic content in MAP extracts makes them good candidates as food additives; however, their concentration decreases during food processing since they are very sensitive to heat, oxygen, and light [4]. Furthermore, despite their claimed health-promoting properties, only a small part of them is absorbed into the gastrointestinal tract, probably because of their low solubility and permeability. In addition, polyphenols confer an astringent and bitter taste to food, which makes them undesirable for most consumers, limiting their use as food additives [5].

Therefore, natural sweeteners such as erythritol or honey have been employed to enhance the antioxidant properties of MAP extracts, as well as to mask the astringent and bitter taste of polyphenols [6,7]. Erythritol is a simple sugar alcohol that is widely distributed in nature. It occurs as a metabolite or storage compound in seaweeds and fungi and is also contained in common fruits, such as melons, grapes, and pears. It can be safely used as a non-cariogenic sweetener in foods, as it cannot be fermented by bacteria that cause dental caries. Erythritol also possesses a low calorie content and glycemic index, which makes it interesting for diabetics [8]. In the study by den Hartog et al. [9], erythritol was shown to be an excellent hydroxyl radical (HO·) scavenger, acting as an antioxidant in vivo and also protecting against hyperglycemia-induced vascular damage. The valuable properties of erythritol mixtures with polyphenols has been also well documented by Nowicka and Wojdyło [7], who studied erythritol addition to sour cherry puree. According to their results, erythritol addition positively affected the antioxidant capacity of sour cherry puree, maintaining its total polyphenols and antioxidant capacity for storage duration up to six months. 

The antioxidant capacity of honey, as well as its high content in polyphenols, has been extensively reviewed [10], but the co-presence of honey in MAP mixtures has been studied to a lesser extent [11,12]. Some researchers have taken the aforementioned advantages of honey and MAPs by mixing honey with various kinds of teas in order to produce appealing beverages for consumers. In the study by Bodor et al. [13], green tea flavored with honeydew honey enhanced its total phenolic content (TPC) from 1200 mg/L to 1500 mg/L, whereas black tea did not enhance its phenolic content probably due to the thermal degradation of polyphenols. On the contrary, Tomczyk et al. [6] found that the combined effect of honey and mulberry leaves resulted in a 10-fold increment in the final antioxidant capacity in the resulting product, highlighting the beneficial effect of honey in biofunctional mixtures.

The efficient incorporation of bioactive constituents inside new food matrices is more than necessary to create new products according to consumers demands. Encapsulation is a promising approach for the protection of bioactive constituents from various environmental stresses, ensuring high bioavailability and stability, whereas it can be also applied to mask unwanted flavor or taste of the core material [14]. To achieve this, one material is entrapped or coated with another material in order to be protected against adverse conditions and nutritional deterioration. The coated one is called the core or active material and the surrounding one is the coating or carrier material [15]. 

Many encapsulation methods have been described in the literature, among which some have been successfully applied in plant polyphenols [16]. Spray drying is the production of highly dispersed powders from a fluid feed by evaporating the solvent after its contact with a heated gas. This method is widely used in the food industry, mainly because of its low operational cost. However, the high temperatures (160–220 °C) applied during drying to facilitate water evaporation are detrimental to plant polyphenols and can reduce their antioxidant potential in food matrices. Nevertheless, the use of specific coating materials can reduce the adverse effects of temperature [16]. To further improve the stability of plant polyphenols in food products, other techniques such as freeze drying have been introduced [17]. Freeze drying, also known as lyophilization, is the process of removing ice or other frozen solvents from a material through sublimation and the removal of bound water molecules by desorption. Freeze drying has the advantage of keeping the product temperature low enough during the process to avoid changes in the dried product appearance and characteristics. However, this method is a very time-consuming, energy-demanding, and cost-intensive process due to its long drying times and many phase transitions that are uneconomical in food processing [18].

Encapsulation by solvent evaporation method has attracted attention in the food and drug industry due to its characteristics, including use for mild conditions, ease of use and scale-up, lower residual solvent, and no change in the activity of bioactive compounds. Solvent evaporation is usually carried out using a rotary evaporator at mild temperatures. Therefore, it is a suitable method to preserve temperature-sensitive biological products that can be stored at room temperature [19,20]. Gum arabic and inulin are the most frequently used encapsulating carriers, according to many studies that have demonstrated their efficiency in the stabilization of bioactive compounds, delaying their chemical degradation and masking the unpleasant taste [21,22]. Inulin, a storage carbohydrate in chicory roots, is a generic term that covers all types of linear fructans. Inulin cannot be digested in the small intestine, but it travels to the lower gastrointestinal tract and acts as a prebiotic by promoting the growth of beneficial bacteria in the gut. Because of these properties, inulin has been specifically applied to the construction of oral delivery systems in the form of nanoparticles and hydrogels for the localized targeting of certain pharmaceuticals related to colon diseases [23]. Besides inulin’s encapsulating properties, it also presents significantly higher antioxidant activity compared to simple sugars, as well as high tolerance of cooking temperatures and digestion processes [24]. Therefore, inulin is considered a safe food ingredient, and is being added to many foods as a sugar and fat replacement, thus improving texture and spreadability [21,22,25].

Gum arabic is a complex polysaccharide from the stems and branches of *Acacia* species, and has recently earned a noticeable interest in the encapsulation domain because of its high water solubility, emulsifying properties, and low viscosity of the concentrated solutions in comparison with other hydrocolloid gums [25]. Therefore, gum arabic is frequently used as a coating material in encapsulation, minimizing the loss of polyphenols in acidic stomach conditions and also maximizing their release in intestinal conditions, where the absorption of food nutrients occurs [26]. This plant-based hydrocolloid is traditionally known for its therapeutic properties in diseases such as diabetes mellitus, stroke, and hypertension, and its strong antioxidant property is the most documented effect [27].

Since polyphenols’ health effects are more than unquestionable, their incorporation into the human diet is necessary to benefit from their cytoprotective effect through various widely consumed food matrices. A typical example of “comfort” food consumed by the majority of the population is chocolate, which may constitute a good matrix for the addition of bioactive health-promoting compounds. Chocolate spreads enriched with omega-3 fatty acids [28] and soluble and insoluble dietary fibers [29], or formulated with dried grape pomace as a healthy partial substitute for sugar and milk-originated powder [30], have been reported in the literature. Tolve et al. [31] used inulin and maltitol as sugar replacer in healthy chocolate hazelnut spreads fortified with vitamin D and magnesium-calcium carbonate nanoparticles, updating their nutritional value. 

A literature survey revealed that there are limited studies regarding enhancement of MAP aqueous extracts using natural sweeteners or coating materials to develop biofunctional food products by applying the solvent evaporation method [32,33]. Most researchers use spray-drying or freeze-drying techniques in order to concentrate MAP extracts bioactive compounds. 

The aim of this study is the enhancement of the TPC and DPPH radical scavenging activity of MAP aqueous extracts using natural sweeteners (such as erythritol or honey) or coating materials (such as gum arabic or inulin) for the phenolic compound incorporation or encapsulation by applying the solvent evaporation method to produce high-added-value biofunctional food products.

## 2. Materials and Methods

This study aims to enhance the TPC and DPPH radical scavenging activity of MAP aqueous extracts. For this purpose, natural sweetener incorporation or encapsulation using coating materials followed by solvent evaporation were applied to produce biofunctional foods with fortified TPC and DPPH radical scavenging activity.

### 2.1. Plant Materials 

Plant materials were kindly provided by Dioscurides P.C., an MAP-processing company located in Anarrachi (Ptolemaida, Greece). The studied plant materials were:

1. *Melissa officinalis* L. leaves;

2. A mixture (M1) of *Melissa officinalis* L. leaves, *Olea europaea* L. leaves, *Cistus incanus* ssp. *creticus* L. leaves, *Aronia melanocarpa* L. fruits, *Punica granatum* L. peel, and *Crocus sativus* L. petals;

3. A mixture (M2) of *Melissa officinalis* L. leaves, *Cistus incanus* ssp. *creticus* L. leaves and *Punica granatum* L. pomace. 

All plant material was air dried at room temperature, weighted, and milled to a final particle size of 1–3 mm, constituting the solid phase of the extraction process. The recovery of phenolic compounds was carried out in a two-step extraction process, including pulsed electric field extraction for 5 min (custom-made apparatus), followed by a 30 min application of ultrasound (Skymen cleaner JP-060S, Shenzhen, China, 360 W, 40 KHz) using water as an extraction solvent at solid/liquid ratio of 1 g plant material/20 mL water. 

### 2.2. Concentration of MAP Aqueous Extracts for the Preparation of Biofunctional Chocolate Bites

In the aqueous extracts of *Melissa officinalis* L., M1 and M2 mixtures of natural sweeteners (erythritol or honey) at a ratio of 1 g sweetener per 20 mL extract were added and heated over a hot plate (50 °C) under continuous stirring for 3 min. The homogeneous mixture was subsequently concentrated 15-fold in a rotary evaporator (Buchi, Rota vapor R124, Flawil, Switzerland) to remove water (solvent) to a final weight of 1.5 g at 45 °C for 4 h. These temperatures do not affect the functionality of the mixtures [34].

To produce a biofunctional chocolate bite, the concentrated mixtures obtained were mixed with commercial bitter chocolate as the base (85% *w*/*w* cocoa) in a final ratio of 1 g concentrate per 7 g chocolate base in every case. To determine the DPPH radical scavenging activity and total phenolic content, the chocolate bites prepared were extracted with 40% (*v*/*v*) ethanol by simple stirring for 5 min and allowed to stand for 24 h. 

### 2.3. MAP Extract Encapsulation 

The *Melissa officinalis* L. and M1 mixture aqueous extracts were encapsulated with gum arabic or inulin as coating materials at a solid-to-liquid ratio of 1 g coating material/20 mL extract. The resulting liquid was concentrated to remove water (solvent) using a rotary evaporator (Buchi, Rota vapor R124, Flawil, Switzerland) at 45 °C for 4 h and stored in the fridge prior to analysis. To determine the DPPH radical scavenging activity and total phenolic content, the *Melissa officinalis* L. and M1 aqueous extracts encapsulated in gum arabic or inulin were extracted with 40% (*v*/*v*) ethanol by simple stirring for 5 min and allowed to stand for 24 h. 

### 2.4. Physicochemical Analyses

Total phenolic content (TPC) of each mixture was determined by the Folin–Ciocalteu spectrophotometric method, based on ISO 14502-1 [35], with a Shimadzu UV 1900 apparatus. The results are expressed in gallic acid equivalents (GAE), i.e., mg GAE/mL of extract or mg GAE/g chocolate.

The DPPH radical scavenging activity of the functional mixtures or chocolate was based on the DPPH (2,2-diphenyl-1-picrylhydrazyl) protocol described by Blois [36] as modified by Bondet et al. [37] and Brand-Williams et al. [38]. The results are expressed as DPPH radical scavenging activity percentage using Equation (1):

(1) $$DPPH\;radical\;scavenging\;activity\;\%=\left(\frac{A_{blanc}-A_{sample}}{A_{sample}}\right)\%$$

where *A_blank_* is the blank absorbance and *A_sample_* is the sample absorbance.

### 2.5. Statistical Analysis

All extraction trials were carried out in triplicate, every sample was analyzed in duplicate, and the reported results are the average values. Error bars and standard deviation of the values are also included in the respective figure and table.

The TPC and DPPH radical scavenging activity of each experimental group were statistically processed using the average measured values in order to identify whether they exhibited a statistical correlation in terms of linear relation.

## 3. Results and Discussion

### 3.1. Assessment of TPC and DPPH Radical Scavenging Activity of MAP Concentrated Aqueous Extracts and Chocolate Bites in the Presence of Erythritol or Honey

**Figure 1:**
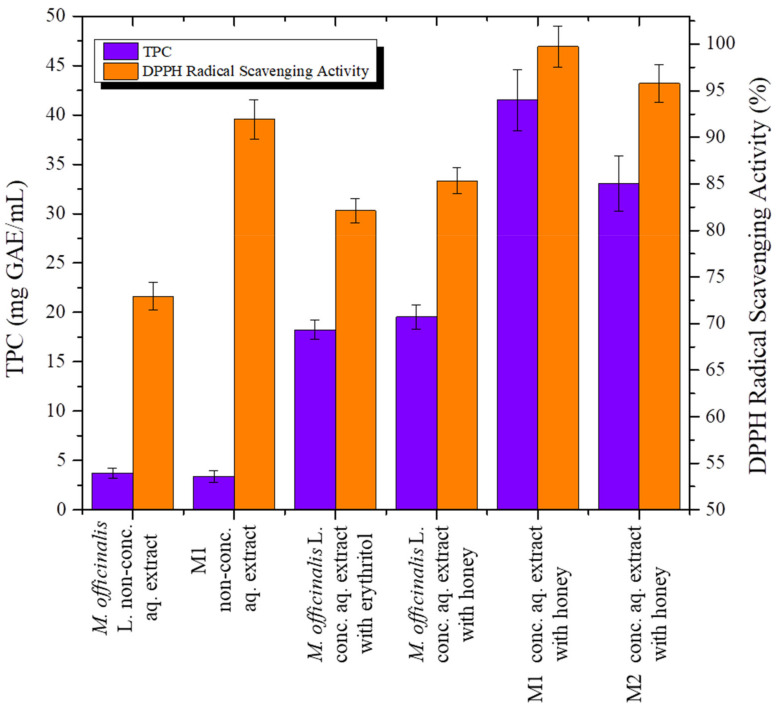
Total phenolic content (TPC) and DPPH radical scavenging activity of medicinal aromatic plant (MAP) concentrated aqueous extracts in the presence of erythritol or honey.

TPC and DPPH radical scavenging activity of *Melissa officinalis* L. concentrated aqueous extracts in the presence of erythritol or honey and M1 and M2 concentrated aqueous extracts in the presence of honey were measured and compared to the respective values of their non-concentrated aqueous extracts. The results are presented in the following Table 1 and Figure 1. 

The calculated correlation coefficient (R^2^ = 0.88) showed that there was a significant linear correlation between TPC and DPPH radical scavenging activity values.

The non-concentrated aqueous extracts of *M. officinalis* L. and M1 showed similar TPC (3.74 mg GAE/mL extract of *M. officinalis* and 3.38 mg GAE/mL extract of M1), whereas the DPPH radical scavenging activity of *Melissa officinalis* L. non-concentrated aqueous extract was 20% lower when compared to M1 non-concentrated aqueous extract. The TPC and DPPH radical scavenging activity of these non-concentrated aqueous extracts were higher than those reported in the literature, and attributed to the applied extraction process of the pulsed electric field/ultrasounds. 

*Melissa officinalis* L. concentrated aqueous extracts with erythritol or honey as natural sweeteners resulted in a 5–6-fold increment in the TPC in its non-concentrated aqueous extract (3.74 mg GAE/mL). Increased TPC and DPPH radical scavenging activity was obtained with the addition of honey (19.53 mg GAE/mL and 85.3%, respectively) compared to erythritol (18.24 mg GAE/mL and 82.1%, respectively) in *Melissa officinalis* L. concentrated aqueous extracts. For this reason, the addition of honey as natural sweetener and solvent evaporation were selected to obtain enhanced TPC and DPPH radical scavenging activity of M1 and M2 concentrated aqueous extracts in order to prepare biofunctional chocolate bites. 

The M1 and M2 concentrated aqueous extracts with honey presented 1.5–2-fold higher TPC (41.52 mg GAE/mL and 33.05 mg GAE/mL, respectively) compared to *M. officinalis* concentrated aqueous extracts with honey. Furthermore, M1 and M2 concentrated aqueous extracts with honey presented greater than 95% DPPH radical scavenging activity (99.7% and 95.7%, respectively). These results highlight the synergistic effect of MAP and natural sweetener bioactive constituents οn TPC and DPPH radical scavenging activity, which were further increased by solvent evaporation application. Synergistic interaction can occur when mixtures of phenolic compounds of different origin exhibit greater effects than each individual mixture of phenolic compounds [12,39,40]. 

In each M1 and M2 concentrated aqueous extract obtained with honey, a chocolate base was added to produce sugar-free biofunctional chocolate bites. The TPC and DPPH radical scavenging activity of the chocolate bites produced were assessed and compared to the TPC and DPPH radical scavenging activity of the chocolate base. The results are presented in Table 2.

The calculated correlation coefficient (R^2^ = 0.99) showed that there was a significant linear correlation between the TPC and DPPH radical scavenging activity values.

The fortified chocolate bite with M1 or M2 concentrated aqueous extracts and honey, increased the TPC of the chocolate base twofold, exhibiting 33.33 mg GAE/g chocolate and 29.48 mg GAE/g chocolate, respectively. The DPPH radical scavenging activity of the chocolate bites was 94.6% and 93.7%, respectively, when M1 or M2 concentrated aqueous extracts with honey were used compared to chocolate base (88.0%). Chocolate bites prepared using M2 concentrated aqueous extract and honey were selected due to their composition, presenting a lower risk of allergic reaction and good organoleptic properties. 

There are few studies that use plant materials of various origins to produce functional food products such as chocolate [41,42]. Acan et al. [30] used grape pomace and achieved a TPC increment in chocolate spread formulations that ranged from 3.415 to 13.754 mg GAE/g chocolate. In their study, higher amounts than 10 g grape pomace/100 g chocolate were not accepted in sensory analysis due to the bitterness of the phenolic compounds, highlighting the necessity for the combined use of polyphenols with alternative sweeteners in the production of functional foods. Büker et al. [43] determined the effects of dried apple pomace addition in chocolate formulations. According to their results, the TPC in chocolate products was between 1.34 mg GAE/g chocolate and 2.42 mg GAE/g chocolate by the addition of 4 g to 20 g of dried apple pomace/100 g chocolate. 

The results of this study show that chocolate bites fortified with MAP mixtures with concentrated aqueous extract and honey as a natural sweetener can be an excellent source of bioactive compounds, revealing their strong potential for successful introduction in the market and increasing antioxidant intake by consumers.

### 3.2. Assessment of TPC and DPPH Radical Scavenging Activity of MAP Concentrated Aqueous Extracts Encapsulated in Gum Arabic or Inulin as Coating Materials

Encapsulation of the bioactive compounds contained in *Melissa officinalis* L. or M1 concentrated aqueous extract using gum arabic or inulin as a coating material was also performed and the TPC and DPPH radical scavenging activity results are presented in the following Table 3. The scope of these experiments was to determine which of the two natural encapsulation materials can be used to produce non-sweet food products.

The calculated correlation coefficient (R^2^ = 0.94) showed that there is a significant linear correlation between TPC and DPPH radical scavenging activity values.

The use of gum arabic or inulin as coating (encapsulation) materials resulted in a fivefold TPC increment in *Melissa officinalis* L. concentrated aqueous extract compared to its non-concentrated aqueous extract (3.74 mg GAE/mL extract). In M1 concentrated aqueous extract, a 10–12-fold higher TPC was observed in the resulting encapsulates (40.37 mg GAE/mL extract using gum arabic and 34.14 mg GAE/mL extract using inulin) compared to its non-concentrated aqueous extract, whereas its DPPH radical scavenging activity successfully approached 99.5% with both encapsulating agents. Encapsulation with gum arabic of both concentrated aqueous extracts of *Melissa officinalis* L. and M1 exhibited higher TPC at 18.37 mg GAE/mL and 40.37 mg GAE/mL, respectively, compared to inulin (16.54 mg GAE/mL and 34.14 mg GAE/mL, respectively). Therefore, gum arabic can be used to encapsulate concentrated aqueous extract and, accordingly, prepare non-sweet biofunctional products with enhanced TPC and DPPH radical scavenging activity. Further studies have to be carried out for the improvement of organoleptic properties using different MAP aqueous extract mixtures.

There are many studies available regarding the use of gum arabic or inulin as encapsulation materials of polyphenols [26,44,45]. Dobrinčić et al. [46] studied the encapsulation of olive leaf extract in gum arabic or inulin and found that gum arabic was more effective as a coating material, preserving to a higher degree the antioxidant properties of the core material compared to inulin. Daza et al. [47] also studied gum arabic and inulin as encapsulation materials of *Eugenia dysenterica* DC, a native fruit of Brazil, by applying spray drying. The TPC ranged between 11 and 31 mg GAE/g DW and between 10 and 29 mg GAE/g DW for samples prepared using gum arabic and inulin, respectively. In the study by Mahdavi et al. [48] the entrapment mechanism of encapsulated molecules is well explained, where gum arabic acts as an excellent film-forming agent due to its chemical structure, also preserving the antioxidant properties of the core material well. 

Our results showed that mixtures of concentrated aqueous plant extracts encapsulated in gum arabic or in the presence of honey as natural sweetener can be strong candidates in the field of active delivery systems, expanding the food industry’s possibilities in the development of new functional products in many food categories.

## 4. Conclusions

Honey or erythritol as natural sweeteners in *Melissa officinalis* L. concentrated aqueous extracts increased its TPC up to sixfold compared to *Melissa officinalis* L. non-concentrated aqueous extracts. The addition of honey had a more profound effect on the TPC and DPPH radical scavenging activity of *M. officinalis* concentrated aqueous extracts, resulting in 19.53 mg GAE/mL and 85.3%, respectively, compared to erythritol (18.24 mg GAE/mL and 82.1%, respectively). Honey as a natural sweetener in MAP concentrated aqueous extract mixtures increased the TPC up to twofold compared to *Melissa officinalis* L. concentrated aqueous extracts with honey.

Chocolate bites fortified with MAP mixtures of concentrated aqueous extracts and honey as natural sweetener can be an excellent source of bioactive compounds, increasing the TPC up to twofold comparing to chocolate base, approaching 33.00 mg GAE/g chocolate and 94% of DPPH radical scavenging activity. The combined use of honey and plant polyphenols in biofunctional foods can become a very promising strategy in the field of high-added-value product development, combining high antioxidant activity with the desired level of sweetness and flavor.

The use of gum arabic as a coating material resulted in a fivefold TPC increase (18.37 mg GAE/mL) in *Melissa officinalis* L. concentrated aqueous extracts compared to its non-concentrated aqueous extracts. In MAP concentrated aqueous extract mixtures, a 12-fold higher TPC was observed in the resulting encapsulate with gum arabic compared to its non-concentrated aqueous extract (3.38 mg GAE/mL). AC successfully approached 99.5%, showing that MAP concentrated aqueous extract mixtures in gum arabic as a coating material can be a strong candidate in the field of active delivery systems, expanding the food industry’s possibilities in the development of new products.

## Figures and Tables

**Table 1:** Total phenolic content (TPC) and DPPH radical scavenging activity of medicinal aromatic plant (MAP) concentrated aqueous extracts in the presence of erythritol or honey.

Sample	TPC (mg GAE/mL)	DPPH Radical Scavenging Activity (%)
*M. officinalis* L. non-concentrated aqueous extract	3.74 ± 0.1	72.9 ± 0.5
M1 non-concentrated aqueous extract	3.38 ± 0.2	91.9 ± 0.3
*M. officinalis* L. concentrated aqueous extract with erythritol	18.24 ± 0.3	82.1 ± 0.4
*M. officinalis* L. concentrated aqueous extract with honey	19.53 ± 0.2	85.3 ± 0.5
M1 concentrated aqueous extract with honey	41.52 ± 1.0	99.7 ± 0.3
M2 concentrated aqueous extract with honey	33.05 ± 1.2	95.7 ± 0.3

**Table 2:** TPC and DPPH radical scavenging activity of chocolate base and chocolate bites fortified with M1 or M2 concentrated aqueous extracts and honey.

Sample	TPC (mg GAE/g Chocolate)	DPPH Radical Scavenging Activity (%)
Chocolate base	15.39 ± 1.2	88.0 ± 1.2
Chocolate bite fortified with M1 concentrated aqueous extract and honey	33.33 ± 0.8	94.6 ± 0.7
Chocolate bite fortified with M2 concentrated aqueous extract and honey	29.48 ± 1.4	93.7 ± 0.5

**Table 3:** TPC and DPPH radical scavenging activity of *Melissa officinalis* L. and M1 concentrated aqueous extracts encapsulated in gum arabic or inulin.

Sample	TPC (mg GAE/mL)	DPPH Radical Scavenging Activity (%)
*M. officinalis* L. concentrated aqueous extract encapsulated in gum arabic	18.37 ± 1.4	80.2 ± 1.1
*M. officinalis* L. concentrated aqueous extract encapsulated in inulin	16.54 ± 0.9	81.6 ± 0.9
M1 concentrated aqueous extract encapsulated in gum arabic	40.37 ± 0.8	99.5 ± 0.8
M1 concentrated aqueous extract encapsulated in inulin	34.14 ± 1.1	99.5 ± 0.7

## Data Availability

Not applicable.
